# Study on the Effect of SBS/HVA/CRM Composite-Modified Asphalt on the Performance of Recycled Asphalt Mixtures

**DOI:** 10.3390/polym16223226

**Published:** 2024-11-20

**Authors:** Haoming Li, Hongkui Wang, Junning Lin, Jiangang Yang, Yuquan Yao

**Affiliations:** 1 CCCC First Highway Consultants Co., Ltd., Xi’an 710075, China; eroslm000@163.com; 2 Foshan Guangming Expressway Co., Ltd., Foshan 528500, China; 13686513166@163.com; 3 CCCC Fourth Harbor Engineering Co., Ltd., Guangzhou 510230, China; 13544362488@163.com; 4 School of Civil Engineering and Architecture, East China Jiaotong University, Nanchang 330013, China; yaoyuquanchd@outlook.com

**Keywords:** recycled asphalt mixture, composite-modified asphalt, optimal asphalt content, road performance

## Abstract

To investigate the feasibility of composite modification techniques in improving the performance of recycled asphalt mixtures, in this study, the high-viscosity agent (HVA) and crumb-rubber materials (CRM) were used to modify asphalt with a styrene-butadiene-styrene block copolymer (SBS), in order to prepare SBS-HVA and SBS-CRM composite-modified asphalts. The virgin asphalt mixtures, as well as three asphalt types of recycled asphalt mixtures with 50% reclaimed asphalt pavement (RAP) content, were designed. The optimal asphalt content of the four types of asphalt mixtures was analyzed, and the rutting test, the asphalt bond strength test, the moisture-induced sensitivity test, and the low-temperature cracking resistance test were conducted to investigate the performance of the four types of asphalt mixtures. The results showed that the higher the asphalt kinematic viscosity, the higher the optimum asphalt content of the asphalt mixtures under the same air voids. HVA significantly improves the adhesion between SBS-modified asphalt and aggregate under dry conditions, while SBS-CRM composite-modified asphalt performs similarly to SBS-modified asphalt. Before and after water immersion, the degree of pull-out strength decay between the asphalts and aggregates follows the sequence of SBS-CRM- > SBS- > SBS-HVA-modified asphalts. Additionally, the residual pull-out work follows the sequence of SBS-HVA- > SBS-CRM- > SBS-modified asphalt. SBS-CRM composite-modified asphalt can significantly improve the moisture sensitivity of recycled asphalt mixtures, as well as low-temperature cracking resistance, while SBS-CRM composite-modified asphalt only improves the low-temperature cracking resistance of recycled asphalt mixtures, and does not improve the moisture sensitivity. Based on the results, it is recommended to select the appropriate composite modification method based on the climate and loading conditions, to maximize the value of asphalt, and to achieve sustainable and durable pavement.

## 1. Introduction

As environmental protection and resource recycling gain increasing attention, asphalt pavement recycling has become a key technique in the field of highway maintenance and construction. The use of reclaimed asphalt pavement (RAP) offers significant benefits, such as conserving natural resources, lowering construction costs, and minimizing the environmental impact of waste asphalt. However, the performance of recycled asphalt mixtures is affected by several factors, particularly the aging of the asphalt in RAP, which leads to reduced durability and mechanical properties compared to virgin asphalt mixtures [1,2,3]. Consequently, developing effective methods to enhance the performance of recycled asphalt mixtures has emerged as a prominent research focus.

Research indicates that styrene-butadiene-styrene block copolymer (SBS)-modified asphalt is widely used in asphalt mixture production. However, recycled asphalt mixtures incorporating SBS-modified asphalt often exhibit insufficient performance and durability [4,5,6]. Kaseer et al. [7] reported that recycled asphalt mixtures with high RAP and recycled asphalt shingles (RAS) content are stiffer and more brittle, making them more prone to cracking. Han et al. [8] found that higher RAP content reduces the low-temperature and fatigue properties of recycled asphalt mixtures, while the moisture stability first improves and then declines. Li et al. [9] found that the fatigue performance of recycled asphalt mixtures decreased with increasing RAP content, and the use of warm-mix asphalt technology will also reduce the fatigue performance of recycled asphalt mixtures. Ai et al. [10] reported that the rutting resistance of recycled asphalt mixtures was improved with an increase in RAP content, while the low-temperature cracking resistance, as well as the moisture stability, would deteriorate. To enhance the performance of recycled asphalt mixtures, rejuvenators are essential in mitigating the effects of aged asphalt in RAP. Kuang et al. [11] found that low-viscosity rejuvenators can restore the water stability and fatigue performance of recycled asphalt mixtures to levels comparable to virgin asphalt mixtures. While the rutting resistance will be reduced, it still meets the specification requirements. Im et al. [12] investigated the effects of the rejuvenator dosage on the high-temperature stability and low-temperature cracking performance of recycled asphalt mixtures. The rejuvenator can reduce the high-temperature stability of recycled asphalt mixtures, but can improve the low-temperature cracking resistance. A balanced high- and low-temperature performance can be achieved by optimizing the rejuvenator dosage.

However, the effectiveness of rejuvenators is limited. To further enhance the performance of recycled asphalt mixtures, many scholars have conducted extensive research on asphalt composite modification to improve both the properties of modified asphalt and the road performance of recycled asphalt mixtures. Ting et al. [13] enhanced the phase stability of SBS-modified asphalt and improved its antioxidant aging ability by adding methylene diphenyl diisocyanate (MDI)-based additives to SBS-modified asphalt. Sun et al. [14] incorporated styreneic methyl copolymers (SMCs) into SBS-modified asphalt to create a composite-modified asphalt, which showed better high- and low-temperature properties than SBS-modified asphalt. Liu et al. [15] studied the properties of composite-modified asphalt composed of desulfurized rubber powder and SBS, and found that the desulfurized rubber powder improves high-temperature performance, but reduces thermal stability. Meng et al. [16] reported that soybean bio-asphalt can improve the rutting factor and deformation recovery rate of SBS-modified asphalt. Hu et al. [17] investigated the rheological properties of high-viscosity-modified asphalt. Shi et al. [18] studied the fatigue performance of composite-modified asphalt using crumb-rubber materials (CRM) from waste tires and SBS, and found that the composite-modified asphalt had a better fatigue performance than SBS-modified asphalt. Therefore, the composite modification technique can improve the asphalt mixture properties by enhancing the asphalt properties. High-viscosity agent (HVA) and CRM are commonly used materials for asphalt modification [19,20]. However, limited research has focused on evaluating the performance of composite-modified asphalt prepared using HVA, CRM, and SBS in recycled asphalt mixtures.

This study mainly explores the feasibility of composite modification technology in improving the performance of recycled asphalt mixtures. To this end, two modifiers of HVA and CRM were selected with SBS-modified asphalt to prepare SBS-HVA, SBS-CRM composite-modified asphalt, the three modified asphalt types of recycled asphalt mixes with 50% RAP content, as well as the virgin asphalt mixture. The optimum asphalt content of different types of asphalt mixtures was analyzed under the same air voids; furthermore, the rutting tests, water sensitivity tests, and low-temperature cracking resistance tests were conducted to analyze the performance of different asphalt mixtures. The research findings offer valuable insights into the use of composite-modified asphalt in recycled asphalt mixtures, offering significant potential for promoting the application of high-RAP blended recycled asphalt mixtures.

## 2. Materials and Methods

### 2.1. Raw Materials

In this study, the raw materials include RAP, virgin aggregate, mineral power, SBS-modified asphalt, rejuvenator, HVA, and CRM.

#### 2.1.1. RAP

**Figure 1:**
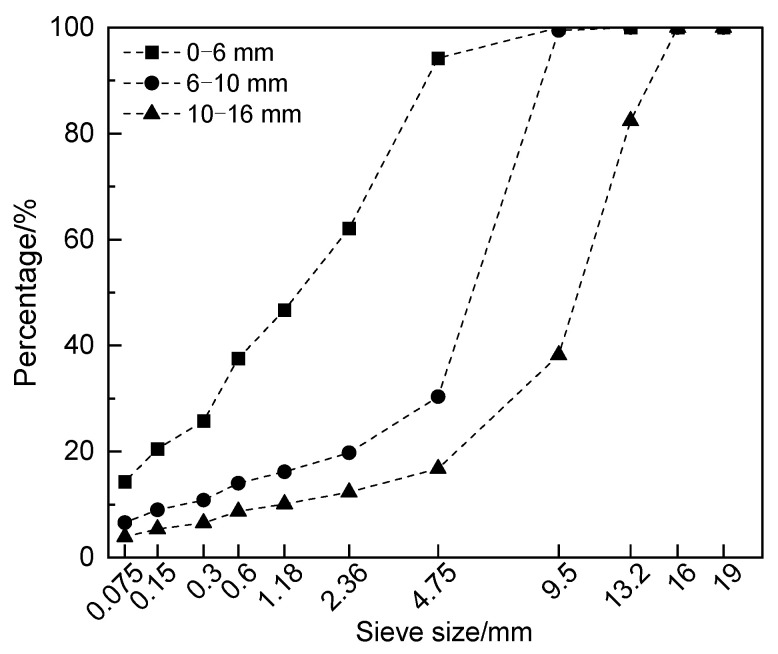
Test results of aggregate gradation in RAP.

RAP was derived from an AC-20 asphalt mixture in the middle surface layer of asphalt pavement, which was obtained by using cold milling equipment, and was divided into three types—0~6 mm, 6~10 mm, and 10~16 mm—of crushing and screening processes. According to the Test Specification for Asphalt and Bituminous Mixtures in Highway Engineering (JTG E20-2011) [21], the centrifugal separation method was used to separate the aggregate and asphalt in RAP. The asphalt content of the three RAP types was 7.35%, 3.68%, and 2.33%, respectively, and the aggregate gradation of RAP was tested using the sieving method, as shown in Figure 1. In addition, the aged asphalt in the aged asphalt solution obtained by centrifugal separation was separated from the solvent by rotary evaporation. The test results of the physical properties of the RAP, the aggregate in the RAP, and the aged asphalt are shown in Table 1. The test results meet the requirements of the specifications [22,23].

#### 2.1.2. Virgin Aggregate and SBS-Modified Asphalt

The virgin aggregate is limestone, according to the Test Methods of Aggregate for Highway Engineering (JTGE42-2005) [24], and the test results of the physical property indexes of new aggregate and mineral powder are shown in Table 2. The SBS-modified asphalt physical performance index test results are shown in Table 3, according to the Standard Test Methods of Bitumen and Bituminous Mixtures for Highway Engineering (JTG E20-2011) [21]. Test results of virgin aggregate and SBS-modified asphalt are to meet the specification requirements.

#### 2.1.3. Rejuvenator

The RA-102 commercial rejuvenator was used in this study, and the basic performance index test results are shown in Table 4. When the dosage of the rejuvenator is 5% of the mass ratio of aged asphalt, the penetration index of regenerated asphalt can meet the requirements of virgin asphalt.

#### 2.1.4. HVA and CRM

**Figure 2:**
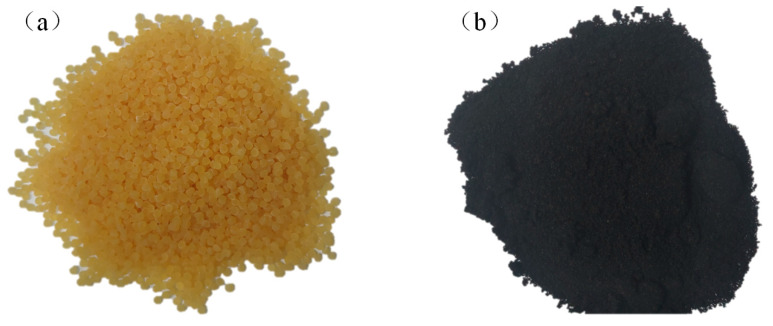
Morphological characteristics of materials. (**a**) HVA, (**b**) CRM.

High-viscosity agent (HVA) and crumb-rubber materials (CRM) were added to SBS-modified asphalt for the preparation of SBS-HVA and SBS-CRM composite-modified asphalt. The HVA was obtained from Shandong China Europe Road and Bridge Group Co., Ltd., (Jinan City, Shandong Province, China) while the CRM, with a particle size of 40 mesh, was derived from recycled trolley tires. The morphological characteristics of the HVA and CRM are presented in Figure 2.

### 2.2. Specimen Preparation

#### 2.2.1. Preparation of Composite-Modified Asphalt

SBS-HVA and SBS-CRM composite-modified asphalts were prepared using HVA, CRM, and SBS-modified asphalt. The preparation process for SBS-HVA composite-modified asphalt is as follows: SBS-modified asphalt was heated to a fully fluid state at 175 °C. Then, 8% HVA (by mass of SBS-modified asphalt) was added, and the composite-modified asphalt was sheared at 400 rad/min for 10 min, 2000 rad/min for 60 min, and 5000 rad/min for another 60 min, at a 180 °C heating temperature. Finally, the composite-modified asphalt was held at 175 °C in the oven for 30 min to complete the process. The preparation process for SBS-CRM composite-modified asphalt is as follows: SBS-modified asphalt was heated to a fully fluid state at 175 °C. Subsequently, 15% CRM (by mass of SBS-modified asphalt) was added. The composite-modified asphalt was sheared at 400 rad/min for 10 min, 5000 rad/min for 20 min, and 5000 rad/min for 30 min, at a 180 °C heating temperature. Finally, the asphalt was held at 175 °C in the oven for 2 h to obtain the SBS-CRM composite-modified asphalt. The test results of the physical properties of the two composite-modified asphalts are shown in Table 5.

#### 2.2.2. Preparation of Recycled Asphalt Mixture

**Figure 3:**
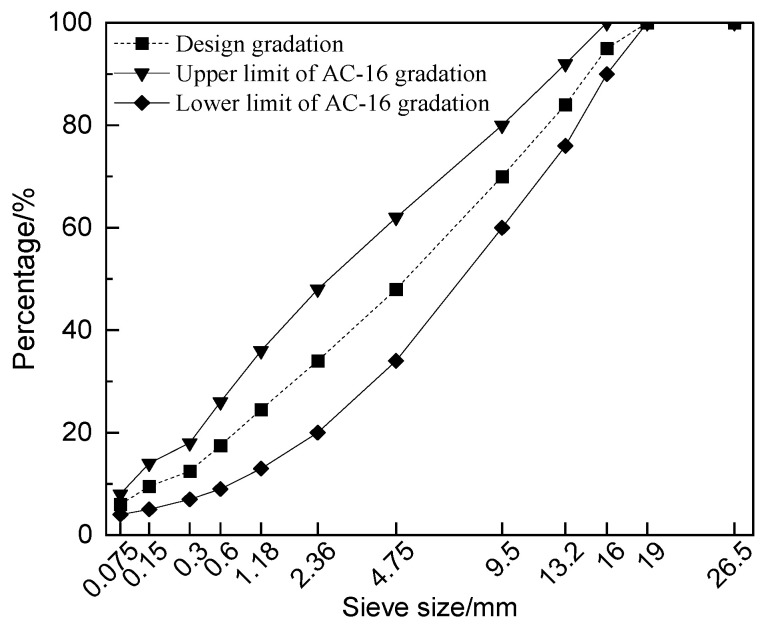
Design gradation of recycled asphalt mixture.

AC-16 recycled asphalt mixtures with 50% RAP content were designed, with the gradation shown in Figure 3. Four types of asphalt mixtures were prepared, as follows: (1) 0% RAP SBS-modified asphalt mixture, (2) 50% RAP SBS-modified asphalt mixture, (3) 50% RAP SBS-HVA composite-modified asphalt mixture, and (4) 50% RAP SBS-CRM composite-modified asphalt mixture. During the preparation of the recycled mixtures, the RAP was preheated to 130 °C for no more than 2 h to minimize further aging of the asphalt in the RAP [25]. The new aggregate was heated to 190 °C. The heating temperatures for the SBS-modified asphalt, the SBS-HVA composite-modified asphalt, and the SBS-CRM composite-modified asphalt were set to 170 °C, 180 °C, and 180 °C, respectively. In the preparation of the 0% RAP SBS-modified asphalt mixture, the new aggregate was heated to 175 °C.

After each material of the recycled asphalt mixture reaches the designated preheating temperature, the mixing process begins. For the 50% RAP asphalt mixture, the RAP is first mixed with the rejuvenator for 60 s, then the virgin aggregate and asphalt are added and mixed for 60 s, and finally, the mineral powder is added and mixed for 60 s to complete the process. For the 0% RAP asphalt mixture, the virgin aggregate is mixed for 60 s, followed by the addition of asphalt for 60 s, and lastly, the mineral powder is incorporated and mixed for 60 s to complete the process. The mixing temperature for all types of asphalt mixtures was 165 °C.

To evaluate the application effect of composite-modified asphalt, the road performances of asphalt mixtures with varying asphalt types and RAP contents under the same air voids were compared. Therefore, in this study, three asphalt contents (3.8%, 4.3%, and 4.8%) were designed for each of the four types of asphalt mixtures, and the optimal asphalt content for each mixture was determined at the 4.0% air voids. Subsequently, four types of asphalt mixtures were prepared separately, using the optimum asphalt content for road performance evaluation.

### 2.3. Experimental Methods

#### 2.3.1. Marshall Test

The Standard Marshall Test was used to mold specimens for four types of asphalt mixtures at a temperature of 155~160 °C. The molded specimens were tested for bulk density, air void (VV), percent voids in mineral aggregate in asphalt mixture (VMA), percent voids in mineral aggregate that are filled with asphalt in asphalt mixture (VFA), Marshall stability (MS), and flow value. These tests helped to determine the optimal asphalt content for different types of asphalt mixtures.

#### 2.3.2. Rutting Test

**Figure 4:**
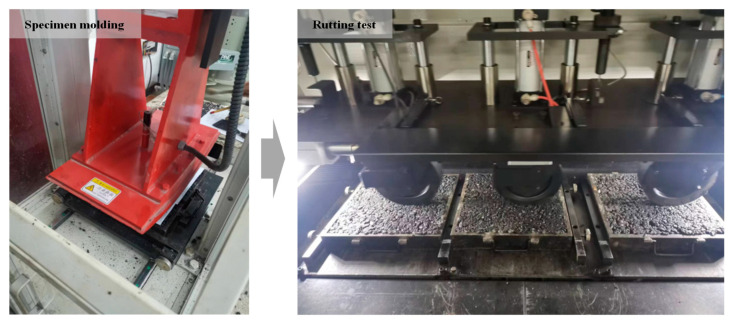
Rutting test process.

Rutting tests were conducted to evaluate the deformation resistance of asphalt mixtures at high temperatures, and to assess their high-temperature stability. Three plate specimens (300 mm in length, 300 mm in width, and 50 mm in height) for each type of asphalt mixture, prepared at the optimal asphalt content, were molded using a wheel mill-forming machine. The rutting test was performed at a temperature of 60 °C, and the average test result from the three specimens was taken as the representative value. The dynamic stability index was used to assess the high-temperature stability of the asphalt mixtures, and the dynamic stability was calculated using Equation (1). The test procedure is illustrated in Figure 4.

(1) $$DS=\frac{\left(t_{2}-t_{1}\right)\times N}{d_{2}-d_{1}}$$

where *DS* represents dynamic stability, cycles/mm. *t*_1_ denotes the wheel milling time at 45 min, and *t*_2_ refers to the wheel milling time at 60 min. *d*_1_ and *d*_2_ correspond to the deformation at 45 min and 60 min, respectively, mm. *N* represents the milling speed, which is typically 42 cycles/mm.

#### 2.3.3. Moisture Sensitivity Test

The moisture sensitivity of composite-modified asphalt was evaluated for both the asphalt binder and the recycled asphalt mixtures. The asphalt bond strength test was used to assess the asphalt binder, while the moisture-induced sensitivity test (MIST) was employed to evaluate the recycled asphalt mixtures.

(1) Asphalt bond strength test

**Figure 5:**
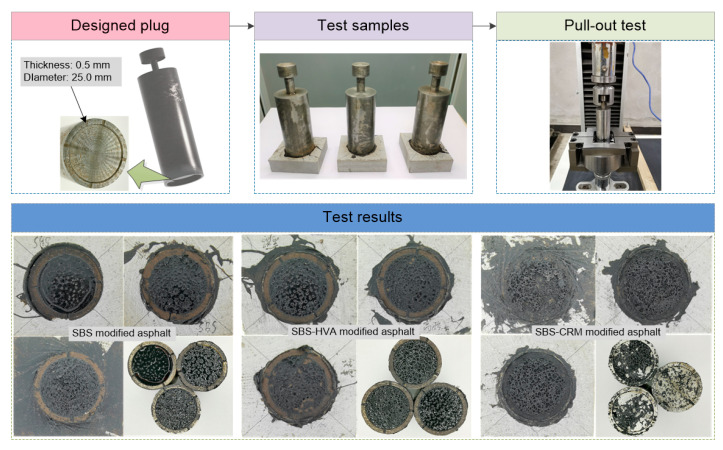
Test process of pull-out test.

The bond strength between the asphalt and aggregate was further evaluated using the pull-out test [26]. The thickness and diameter of the asphalt film in the pull-out test were 0.5 mm and 25 mm. Six parallel specimens were tested in each group, with the following sample preparation process: 0.5 g of asphalt binder was taken using the mold for DSR sample preparation, and then the plug and limestone were placed in an oven at 60 °C for at least 1 h. Next, 0.5 g of asphalt binder was placed in the center of the plug, and the plug was quickly placed on the limestone. Lastly, 5 kg iron was placed on top of the 6 pullers, and they were then moved to an oven for 1.5 h at the test temperature. During the pull-out test, the pulling speed was 10 µm/s, and the pulling was stopped when the gap reached 5000 µm. The pulling force during the loading process was collected. The pull-out test process is shown in Figure 5. The study also compared the pull-out properties between the asphalt and aggregate in the presence of water erosion, where the samples were immersed in a water bath at 40 °C for 96 h [26,27]. The adhesion properties between the asphalt and aggregate were tested at 15 °C, and the adhesion characteristics were characterized using the pull-out strength, pull-out work, residual pull-out strength ratio, and residual pull-out work ratio, which can be calculated using Equations (2)–(5) [26,27].

(2) $$P_{pull}=\frac{F_{peak}}{A}$$

where $P_{pull}$ refers to the pull-out strength between the asphalt and aggregate, MPa; $F_{peak}$ refers to the peak of the pull-out force, N; A refers to the cross-sectional area of asphalt, mm^2^.

(3) $$W_{pull}=\frac{S\times V}{T}$$

where $W_{pull}$ refers to the pull-out work between the asphalt and aggregate, mJ; S refers to the integral area of force and the displacement curve during the pull-out test; V refers to the loading rate, mm/s; T refers to the initial thickness of asphalt film, mm.

(4) $$RSR=\frac{P_{pull-wet}}{P_{pull-dry}}$$

where RSR refers to the residual pull-out strength ratio, %; $P_{pull-wet}$ and $P_{pull-dry}$ refer to the pull-out strength before and after water erosion between the asphalt and aggregate, MPa.

(5) $$RWR=\frac{W_{pull-wet}}{W_{pull-dry}}$$

where RWR refers to the residual pull-out work ratio, %; $W_{pull-wet}$ and $W_{pull-dry}$ refer to the pull-out work before and after water erosion between the asphalt and aggregate, mJ.

(2) Moisture-induced sensitivity test

Due to the fact that the AC-16 asphalt mixtures are typically used for surface layers, a moisture-induced sensitivity test was conducted to simulate water washout conditions and evaluate the moisture stability of the asphalt mixtures. The test was performed at a design pressure of 276 kPa and a temperature of 60 °C. Four types of asphalt mixture specimens were prepared, with twenty specimens molded for each type of asphalt mixture under the optimal asphalt content. The specimens were subjected to 0, 1000, 3000, 5000, and 7000 scouring cycles, with four specimens tested for each scouring cycle. After the specimens reached the specified number of scouring cycles, the specimens were placed in a water bath at 15 °C for 2 h, and then the splitting test was conducted. The average test results were used as representative values to analyze the moisture sensitivity of the different asphalt mixtures.

**Figure 6:**
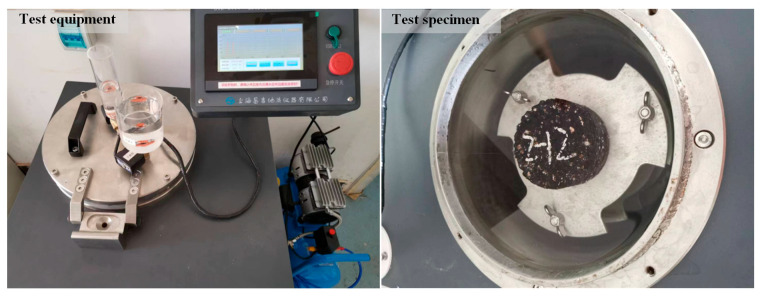
Test process of moisture-induced sensitivity test.

After the specimens reached the specified number of scouring cycles, the water absorption rate, air voids, and split tensile strength of the specimens were measured before and after scouring. The split tensile strength ratio (TSR) was calculated to assess the moisture sensitivity of the different asphalt mixtures. The calculations for split tensile strength and TSR are provided in Equations (6) and (7). The test procedure is shown in Figure 6.

(6) $$TS=\frac{0.006287P_{T}}{h}$$

where *TS* denotes the splitting tensile strength of the asphalt mixture specimen, MPa; *P_T_* represents the splitting strength of the asphalt mixture specimen, N; *h* indicates the height of the asphalt mixture specimen, mm.

(7) $$TSR=\frac{TS_{\mathrm{con}}}{TS_{uncon}}\times 100$$

where *TSR* represents the splitting tensile strength ratio of asphalt mixture specimens, %. *TS_con_* denotes the splitting tensile strength of specimens after dynamic water washout, MPa. *TS_uncon_* refers to the splitting tensile strength of specimens without dynamic water washout, MPa.

#### 2.3.4. Low-Temperature Crack Resistance Test

The semi-circular bending (SCB) test was conducted to evaluate the low-temperature cracking resistance of different types of asphalt mixtures. The SCB specimens had a diameter of 150 mm and a thickness of 50 mm, with a central notch depth of 15 mm and a width of 1.5 mm. For each type of asphalt mixture, four SCB specimens were prepared with the optimum asphalt content. The average of the test results was used as the representative value to characterize the low-temperature cracking resistance of the asphalt mixtures.

**Figure 7:**
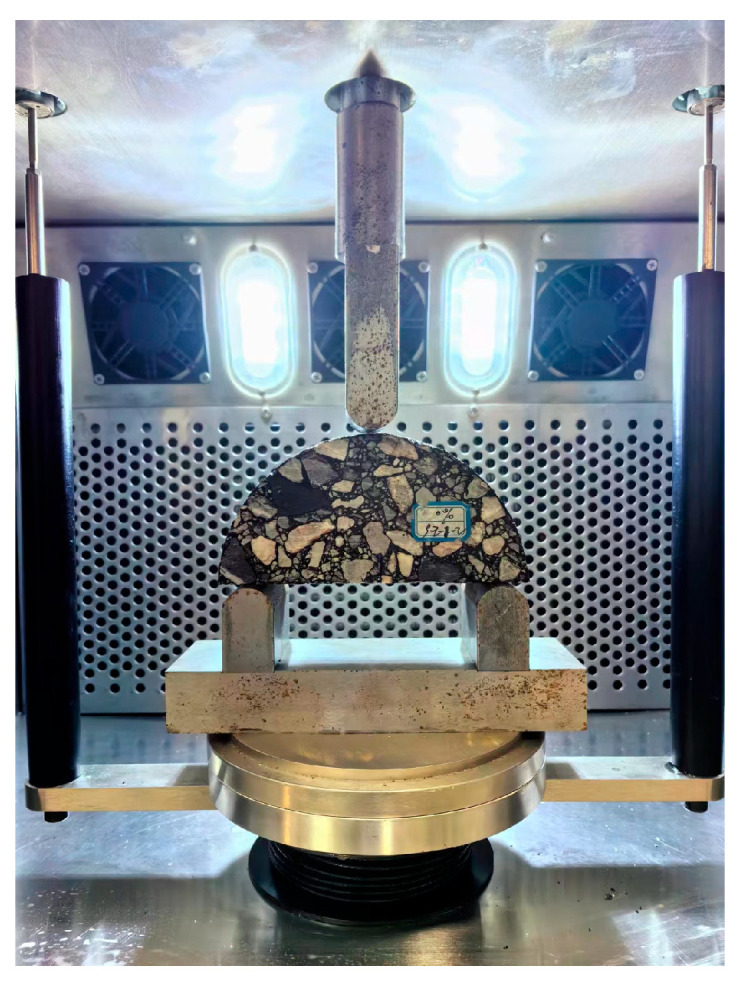
Semi-circular bending test.

The SCB test is typically conducted in the control mode of a crack mouth opening displacement (CMOD). However, this method requires specialized equipment. Feng et al. [28] demonstrated that loading the SCB specimen at a constant rate of 0.02 mm/s ensures stable crack propagation. Therefore, in this study, a constant loading rate of 1.2 mm/min was applied, with the test conducted at a temperature of −10 °C. The test procedure is illustrated in Figure 7.

Based on the energy evaluation criterion, the low-temperature cracking resistance of asphalt mixtures was assessed through peak load, fracture energy, and fracture toughness indices. Fracture energy is calculated as shown in Equation (8). According to fracture mechanics theory, specimens with a pre-cut at the bottom center, which exhibit Mode I cracking behavior, were evaluated using the stress intensity factor (*K*) criterion to assess the damage behavior in the SCB test. The fracture toughness is calculated as shown in Equations (9) to (11).

(8) $$G_{f}=\frac{W_{f}}{(r-a)\times t}\times 10^{6}$$

(9) $$K_{I\;C}=Y_{I\;\;(0.8)}\times \sigma_{0}\times \sqrt{\pi a}$$

(10) $$\sigma_{0}=\frac{P_{c}}{2rt}$$

(11) $$Y_{I\;(0.8)}=4.782+1.219\left(\frac{a}{r}\right)+0.063e^{7.045\left(\frac{a}{r}\right)}$$

where *G_f_* denotes the fracture energy, J/m^2^; *W_f_* denotes the fracture work, expressed using the area enclosed by the peak load and displacement; *r*, *a*, and *t* denote the radius of the SCB specimen, the length of the central notch, and the thickness of the specimen, respectively, mm; *K*_IC_ denotes the fracture toughness, MPa/m; *Y*_I(0.8)_ denotes the standardized stress intensity factor; and $\sigma_{0}$ denotes the stress corresponding to the peak load, MPa.

## 3. Results and Discussion

### 3.1. Determine the Optimal Asphalt Content

The test results for Marshall volumetric indices, Marshall stability, and flow values were obtained for asphalt mixtures with 3.8%, 4.3%, and 4.8% asphalt content. The mixtures tested included the following: a conventional hot-mix SBS-modified asphalt mixture (SBS-V), an SBS-modified asphalt mixture with 50% RAP content (SBS-R), an SBS-HVA composite-modified asphalt mixture with 50% RAP content (SBS-HVA-R), and an SBS-CRM composite-modified asphalt mixture with 50% RAP content (SBS-CRM-R). These results are presented in Table 6. Table 6 illustrates the effects of increasing asphalt content on various asphalt mixtures. As the asphalt content rises, the specific gravity and VFA increase, while the air voids and VMA decrease. However, there is no clear pattern observed for Marshall stability and flow values. The optimum asphalt content for mixtures SBS-V, SBS-R, SBS-HVA-R, and SBS-CRM-R, based on 4.0% air voids, is 4.48%, 4.26%, 4.58%, and 4.71%, respectively.

**Figure 8:**
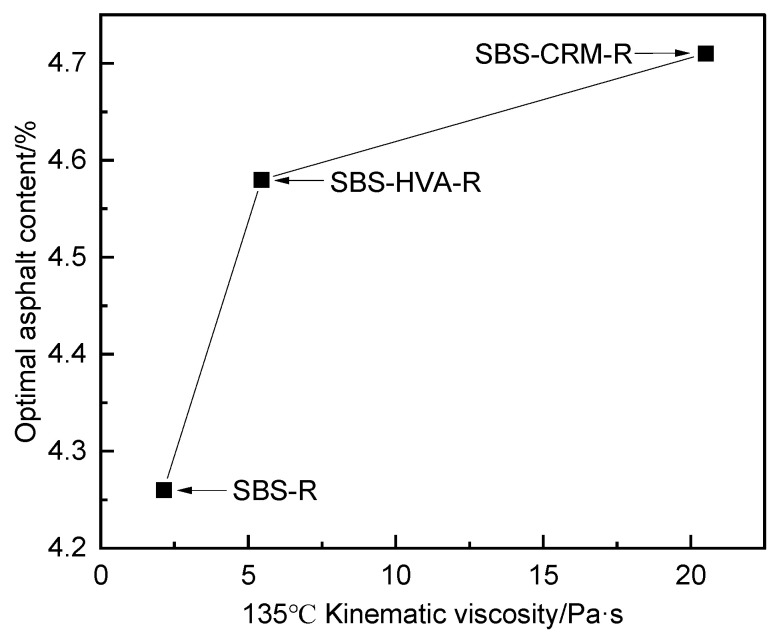
Variation of asphalt kinematic viscosity and optimal asphalt content for recycled asphalt mixture.

Based on the kinematic viscosity test results for the three types of modified asphalt, the changes in kinematic viscosity and the optimal asphalt content for recycled asphalt mixtures were determined, as shown in Figure 8. Figure 8 demonstrates that as the kinematic viscosity of the modified asphalt increases, the optimal asphalt content in the recycled asphalt mixture also increases.

### 3.2. Rutting Test Results

**Figure 9:**
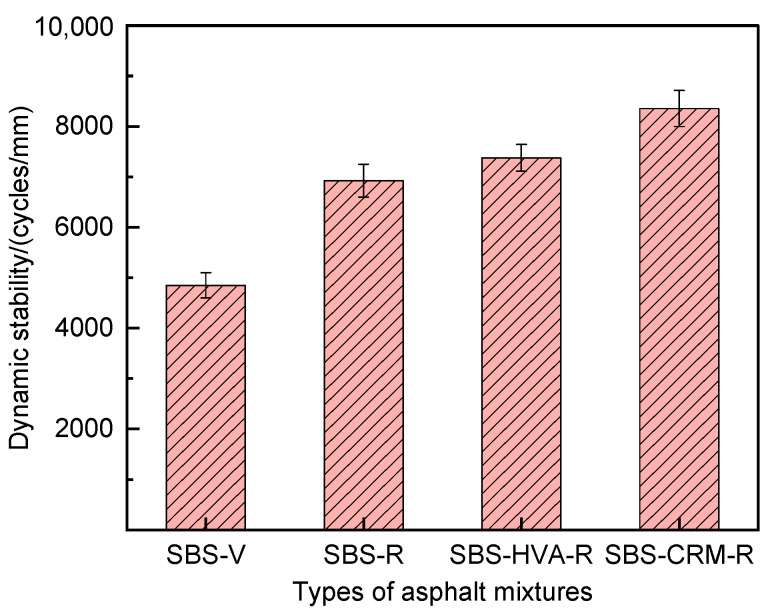
Dynamic stability test results of rutting tests.

The rutting test results for the four types of asphalt mixtures are shown in Figure 9. The dynamic stability of recycled asphalt mixtures with RAP is significantly higher than that of asphalt mixtures without RAP when using the same SBS-modified asphalt, with an increase in dynamic stability of 42.7%. This improvement may be attributed to the aged asphalt in the RAP, which is harder than virgin asphalt, thereby enhancing the recycled asphalt mixture’s resistance to high-temperature deformation [29,30]. With a 50% RAP content, the dynamic stability of SBS-R, SBS-HVA-R, and SBS-CRM-R recycled asphalt mixtures was 6921 cycles/mm, 7378 cycles/mm, and 8357 cycles/mm, respectively. Compared to SBS-R recycled asphalt mixtures, the addition of SBS-HVA and SBS-CRM composite-modified asphalt increased the dynamic stability by 6.6% and 20.7%, respectively.

HVA is a high-viscoelasticity modifier that, when added to SBS-modified asphalt, significantly increases the asphalt’s high-temperature viscosity. This enhancement reduces the mixture’s mobility under high-temperature conditions, making deformation more difficult. In addition, HVA improves the adhesion between the asphalt and aggregates, preventing relative slippage and enhancing the mixture’s structural stability. CRM imparts significant elasticity to the asphalt, and incorporating CRM into the SBS-modified asphalt enhances the mixture’s ability to recover from deformation, effectively resisting plastic deformation at high temperatures. Furthermore, CRM reduces the temperature sensitivity of asphalt, ensuring a more stable performance in high-temperature environments, and minimizing flow deformation caused by temperature fluctuations. Thus, the composite modification technology enhances the high-temperature stability of SBS-HVA and SBS-CRM recycled asphalt mixtures.

### 3.3. Moisture Sensitivity Test Results

#### 3.3.1. Asphalt Binder Bond Strength Test Results

**Figure 10:**
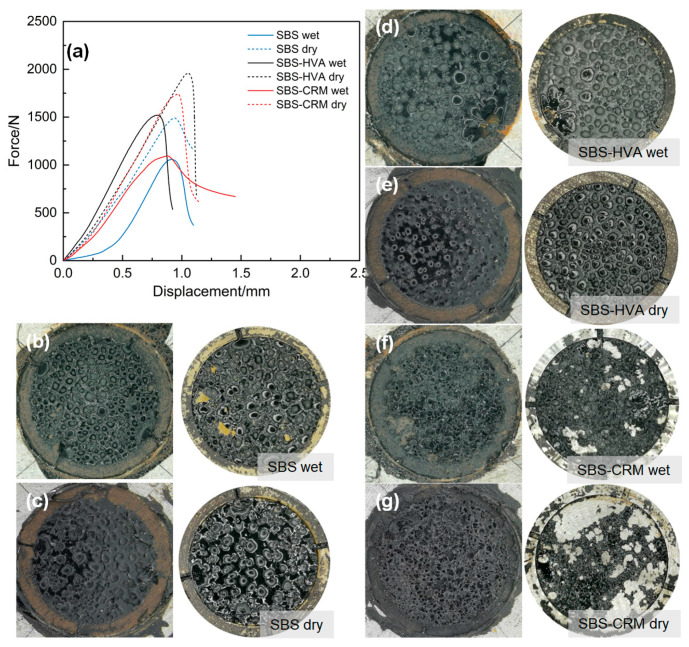
Pull-out test results and the failure image of different asphalt binders: (**a**) force and displacement curves of different asphalt binders; (**b**) failure image of SBS-modified asphalt under wet conditions; (**c**) failure image of SBS-modified asphalt under dry conditions; (**d**) failure image of SBS-HVA-modified asphalt under wet conditions; (**e**) failure image of SBS-HVA-modified asphalt under dry conditions; (**f**) failure image of SBS-CRM-modified asphalt under wet conditions; (**g**) failure image of SBS-CRM-modified asphalt under dry conditions.

The force–displacement variation curves and failure modes in different asphalt pull-out tests are presented in Figure 10.

As shown in Figure 10a, the maximum tensile force of the different asphalt binders was higher before immersion than after. The displacement when the maximum tensile force occurred was between 0.8 and 1.0 mm. This suggests that water damage reduces the adhesion of asphalt to the aggregate. Previous studies [31,32,33] have demonstrated three types of failure in asphalt pull-out tests, namely adhesive, adhesive/cohesive, and cohesive failure. As shown in Figure 10b,c, under dry conditions, the SBS-modified asphalt failed inside the binder, indicating cohesive failure. After water immersion, the failure interface appeared partly on the surface of the aggregate/plug, suggesting both adhesive and cohesive failure. For the results presented in Figure 10d,e, the failure interface of SBS-HVA-modified asphalt appeared inside the asphalt before and after water immersion, indicating cohesive failure. Figure 10f,g illustrate that the failure interface of SBS-CRM-modified asphalt after water immersion appeared inside the asphalt binder, as well as on the surface of the aggregate/plug, which exhibits adhesive/cohesive failure. However, most of the failure interfaces of SBS-CRM-modified asphalt before water immersion were on the surface of the plug, indicating cohesive failure, which could be considered adhesive failure. Overall, water immersion significantly reduces the adhesive properties of the asphalt to the limestone substrate, resulting in adhesive strength that is lower than the cohesive strength (adhesive failure), or close to the cohesive strength (adhesive/cohesive failure).

**Figure 11:**
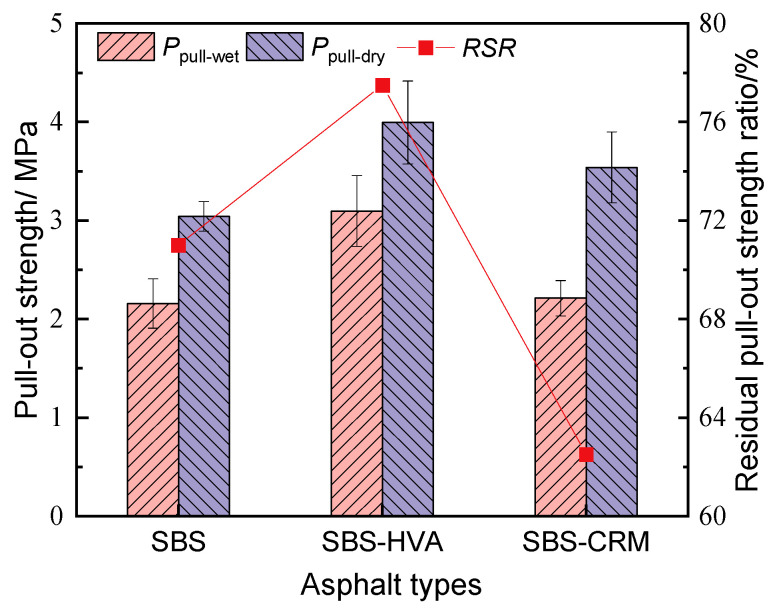
Pull-out strength test results of different asphalts under wet and dry conditions.

Figure 11 illustrates the results of pull-out strengths and residual pull-out strength ratios (RSR) of different types of asphalt before and after water immersion. The pull-out strengths of all three types of asphalt were lower after water immersion than before, indicating that water damage significantly affects the bond between asphalt and aggregates. The pull-out strengths of the three types of asphalt decreased by 22.5% to 37.5% under the effect of water immersion, with the SBS-CRM-modified asphalt exhibiting the smallest RSR, while SBS-HVA had the highest. Moreover, the pull-out strengths of SBS-HVA- and SBS-CRM-modified asphalt were higher than those of SBS-modified asphalt, both before and after water immersion. The pull-out strengths of SBS-HVA-modified asphalt and SBS-CRM-modified asphalt increased by 31.3% and 16.4%, respectively, compared to SBS-modified asphalt without water immersion. After water immersion, the pull-out strengths of SBS-HVA- and SBS-CRM-modified asphalt increased by 43.4% and 2.4%, respectively. Hence, it can be concluded that the water damage resistance order of the three types of asphalt was SBS-HVA- > SBS- > SBS-CRM-modified asphalt.

**Figure 12:**
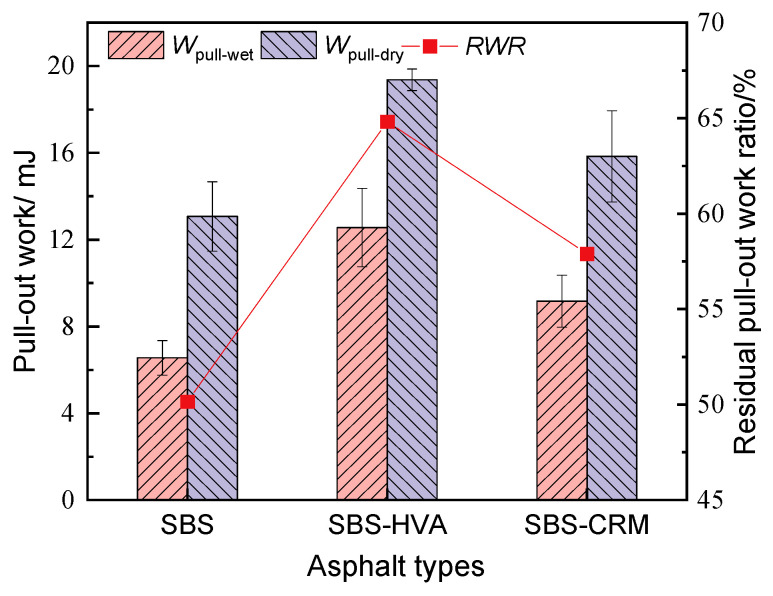
Pull-out work test results of different asphalts under wet and dry conditions.

The pull-out work test results of different asphalt before and after water immersion are presented in Figure 12. The trend in pull-out work before and after water immersion matched the pull-out strength, with SBS-HVA- > SBS-CRM- > SBS-modified asphalt. Before water immersion, the pull-out work for SBS-HVA- and SBS-CRM-modified asphalt showed increases of 48.2% and 21.1%, respectively, compared to SBS-modified asphalt. The pull-out work after immersion increased by 91.5% and 39.9% for SBS-HVA- and SBS-CRM-modified asphalt, respectively. The pull-out work of all three types of asphalt decreased by 35.2% to 49.8% after water immersion. SBS-HVA-modified asphalt had the highest residual pull-out work ratio (RWR), while SBS-modified asphalt had the lowest. Furthermore, the reduction in pull-out work of the three types of asphalt was more significant than that of the pull-out strength, indicating that the impact of water damage on the pull-out work was more significant.

#### 3.3.2. Moisture Stability Test Results

The water absorption, air voids, and splitting tensile strength of the specimens were measured before and after the moisture-induced sensitivity test. The splitting tensile strength ratio was calculated to assess the moisture sensitivity of the four types of asphalt mixtures.

(1) Water absorption

**Figure 13:**
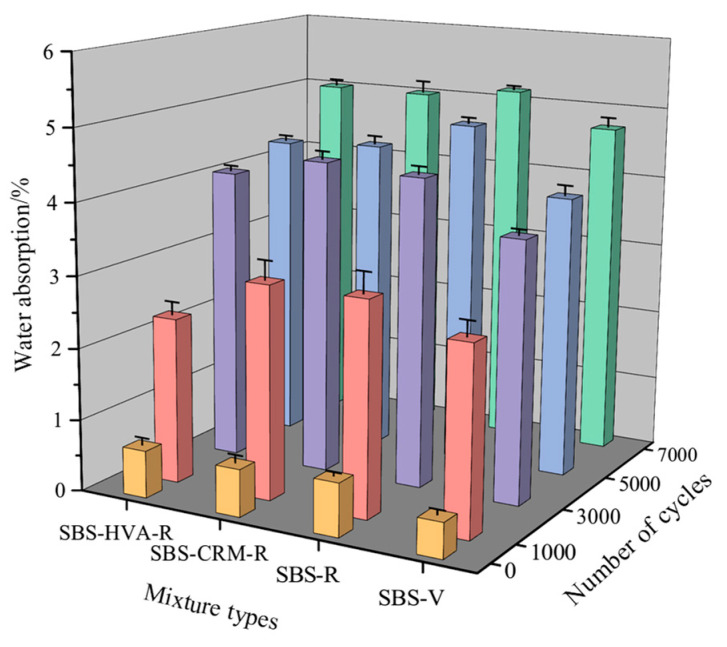
Water absorption of the four types of asphalt mixtures.

Figure 13 shows that the water absorption of the four types of asphalt mixtures increases with the number of scouring cycles, indicating that the increase in scouring cycles damages the internal structure of the asphalt mixture and promotes more water to permeate into the asphalt mixes. The SBS-R asphalt mixture had higher water absorption than the SBS-V asphalt mixture, while the SBS-HVA-R asphalt mixture had lower water absorption than both the SBS-CRM-R and SBS-R asphalt mixtures after the same number of cycles. The growth rate of water absorption of different asphalt mixes under different scouring cycles is shown in Table 7. It can be observed that the water absorption increases significantly during the initial stage of the test (1000 cycle number), and then the growth trend of water absorption gradually decreases with the increase of scouring cycles.

(2) Air voids

**Figure 14:**
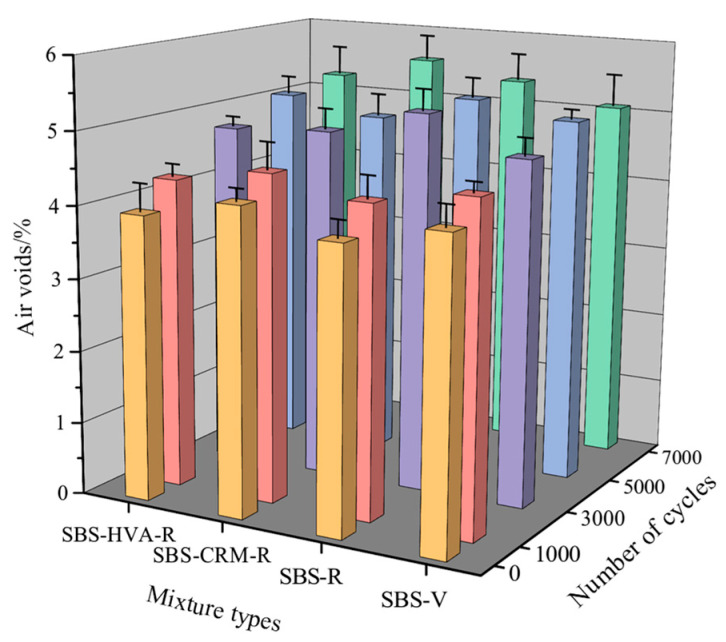
Air voids of the four types of asphalt mixtures.

Figure 14 shows the effect of the number of scouring cycles on air voids in the four types of asphalt mixtures. During the 0 to 7000 cycles, the air voids increased with the number of cycles, but no significant difference in air voids was observed among the four types of asphalt mixes. Table 8 shows that the growth rate of air voids of the four asphalt mixes first increased and then decreased, indicating that early water damage has a significant impact on the variation of air voids in asphalt mixes. The growth rate of air voids in the SBS-V asphalt mixture was lower than that of the asphalt mixture containing 50% RAP, indicating that RAP deteriorates the moisture stability of the asphalt mixture, leading to increased air voids in the asphalt mixture after scouring.

(3) Splitting tensile strength

**Figure 15:**
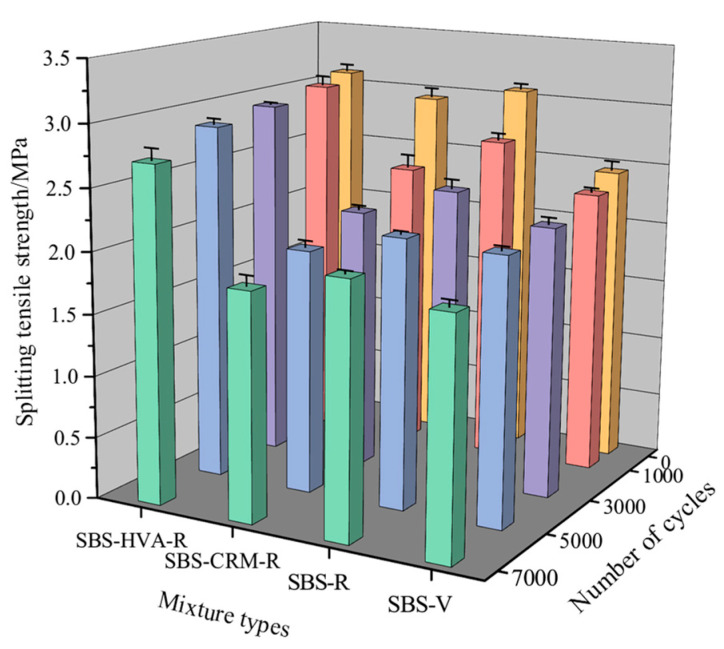
Splitting tensile strength of the four types of asphalt mixtures.

Figure 15 presents the splitting tensile strength results of the four types of asphalt mixtures at various scouring cycles. The splitting tensile strength of all mixtures decreases progressively, with an increasing number of scouring cycles. This decline is primarily due to the dynamic water scouring, which weakens the adhesion between the asphalt and aggregate, leading to reduced splitting tensile properties [34]. The splitting tensile strength of SBS-V asphalt mixtures was lower than that of SBS-R, SBS-CRM-R, and SBS-HVA-R recycled asphalt mixtures, likely due to the presence of aged asphalt in RAP [34]. Table 9 shows that the splitting tensile strength of SBS-V and SBS-HVA-R asphalt mixtures decreased more slowly than that of SBS-R and SBS-CRM-R asphalt mixtures. This suggests that aged asphalt accelerates the reduction in tensile strength for RAP-containing mixtures, while SBS-HVA composite-modified asphalt mitigates this effect. Composite-modified asphalt with an HVA exhibits excellent adhesion properties (as shown in Figure 12) and viscoelasticity (as shown in Figure 8). These characteristics significantly enhance the adhesion between asphalt and aggregate, mitigating the effects of dynamic water scouring and reducing the decay rate of splitting tensile strength. When the number of cycles increased from 5000 to 7000, the rate of tensile strength decline in all four mixtures rose sharply, indicating significant internal structural damage.

(4) Splitting tensile strength ratio

**Figure 16:**
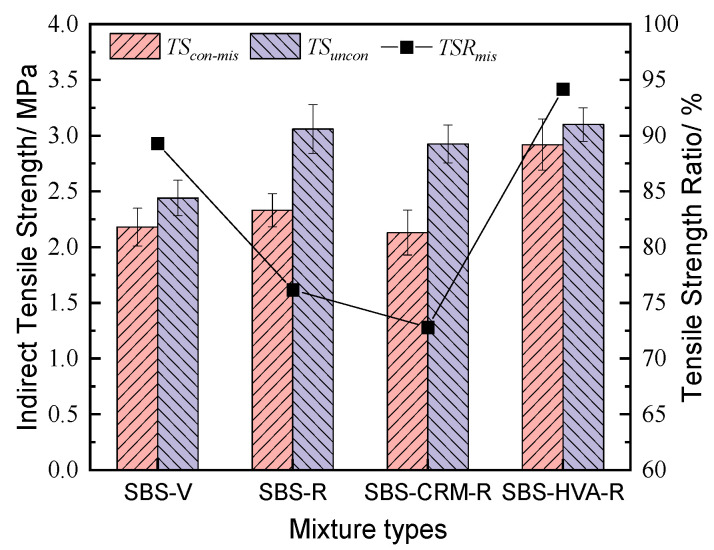
Splitting tensile strength ratio of the four types of asphalt mixtures.

The calculation results of the splitting tensile strength and splitting tensile strength ratio index of different types of asphalt mixture specimens under 3500 scouring cycles are shown in Figure 16. Figure 16 shows that the splitting tensile strength of SBS-V asphalt mixtures, before and after scouring, was lower than that of recycled asphalt mixtures with 50% RAP. However, the splitting tensile strength ratio followed the opposite trend. The splitting tensile strength ratio of SBS-V and SBS-R asphalt mixtures is 89.3% and 76.2%, respectively, with a decrease in 14.7%. This indicates that using RAP can reduce the moisture sensitivity of recycled asphalt mixtures. In addition, the splitting tensile strength ratios of SBS-CRM-R and SBS-HVA-R asphalt mixtures were 72.8% and 94.2%, respectively. Compared to the SBS-R asphalt mixture, SBS-CRM composite-modified asphalt will be detrimental to the moisture sensitivity of the recycled asphalt mixture, while SBS-HVA composite-modified asphalt will significantly improve moisture sensitivity. Furthermore, compared to the SBS-V asphalt mixture, the recycled asphalt mixture using SBS-HVA composite-modified asphalt has higher splitting tensile strength, indicating that SBS-HVA composite-modified asphalt has better moisture sensitivity.

### 3.4. Low-Temperature Crack Resistance Test Results

The results of the low-temperature cracking performance of different types of asphalt mixtures obtained by the SCB test are shown in Table 10. The low-temperature peak load, fracture energy, and fracture toughness of recycled asphalt mixtures with RAP were lower than those of SBS-V asphalt mixtures. This suggests that using RAP reduces the low-temperature cracking performance of recycled asphalt mixtures, mainly because the aged asphalt in the RAP has a poorer low-temperature performance than virgin asphalt.

Compared to the SBS-V asphalt mixture, the peak load of SBS-R, SBS-CRM-R, and SBS-HVA-R decreased by 11.5%, 8.7%, and 6.1%, respectively, the fracture energy decreased by 19.1%, 7.4%, and 9.9%, respectively, and the fracture toughness decreased by 11.6%, 8.8%, and 9.9%, respectively. The low-temperature performance of the recycled asphalt mixture made with SBS-CRM and SBS-HVA composite-modified asphalt was better than that of the mixture made with SBS-modified asphalt. Therefore, composite-modified asphalt can improve the low-temperature performance of recycled asphalt mixtures compared to single-SBS-modified asphalt. However, even with the use of composite-modified asphalt, the low-temperature crack resistance of the recycled asphalt mixture was still lower than that of the SBS-V asphalt mixture. This shows that composite modification has limited effectiveness in improving the low-temperature performance of recycled asphalt mixtures. Thus, it is necessary to improve the performance of recycled asphalt mixtures from the perspective of restoring the low-temperature performance of aged asphalt in RAP.

## 4. Conclusions

This study investigates the effect of composite modification technology on the performance of recycled asphalt mixtures containing 50% RAP. Based on the test results, the main conclusions can be obtained as follows:

(1) The kinematic viscosity of SBS-HVA and SBS-CRM composite-modified asphalts was significantly higher than that of SBS-modified asphalt. Recycled asphalt mixtures with higher asphalt kinematic viscosity required higher optimal asphalt content to meet the 4.0% air void criterion, with SBS-R, SBS-HVA-R, and SBS-CRM-R mixtures having optimal asphalt contents of 4.26%, 4.58%, and 4.71%, respectively.
(2) The dynamic stability of RAP-containing asphalt mixtures prepared with SBS-modified asphalt exceeded that of virgin asphalt mixtures. Mixtures using SBS-HVA and SBS-CRM composite-modified asphalts demonstrated even higher dynamic stability, indicating superior high-temperature performance.
(3) The pull-out strength of SBS-HVA and SBS-CRM composite-modified asphalt was higher than that of SBS-modified asphalt under dry conditions. The interface failure mode was primarily cohesive for SBS-modified and SBS-HVA asphalts, but it was adhesive for SBS-CRM asphalt. Wet conditions reduced the pull-out strength of all three asphalts, negatively impacting adhesion. SBS-HVA asphalt showed the highest pull-out strength ratio, while SBS-CRM asphalt had the lowest. Residual pull-out work ratios ranked as SBS-HVA > SBS-CRM > SBS, with SBS-HVA composite-modified asphalt exhibiting the best moisture resistance.
(4) Dynamic water scouring increased water absorption and air voids while decreasing splitting tensile strength in all asphalt mixtures. The recycled asphalt mixtures showed lower moisture stability than virgin asphalt mixtures. SBS-HVA composite-modified asphalt significantly enhanced moisture stability, whereas SBS-CRM had a minimal effect.
(5) The low-temperature crack resistance of asphalt mixtures containing RAP was lower than that of virgin asphalt mixtures. SBS-HVA and SBS-CRM composite-modified asphalts improved on low-temperature performance compared to SBS-R mixtures, but could not match the performance of virgin mixtures.
(6) Based on performance tests of composite-modified and recycled asphalt mixtures, it is recommended to use 8% HVA and 15% CRM, calculated from the mass of the asphalt, in composite-modified asphalt. Future studies will focus on verifying the feasibility of these experimental results in practical engineering applications.

## Figures and Tables

**Table 1:** Physical properties of RAP.

Material Type	Indicators	Test Results	Test Method [21,23]
RAP	Moisture content, %	0.92	T 0307 and T 0334
	Sand equivalent, %	84.6	
Asphalt in RAP	Penetration, 25 °C, 0.1 mm	34.2	T 0604
	Softening point, °C	68.4	T 0606
	Ductility, 15 °C, cm	14.2	T 0605
	Viscosity, 135 °C, Pa·s	1.73	T 0625
Coarse aggregate	Crushing value, %	11.4	T 0316
	Flakiness and elongation particles, %	13.6	T 0312
Fine aggregate	Angularity	31.7	T 0345

**Table 2:** Physical properties of virgin aggregate and mineral powder.

Aggregate Type	Test Items	Test Results	Test Method [24]
Coarse aggregate	Specific gravity	2.691	T 0304
	Flakiness and elongation particles, %	10.2	T 0312
	Crushing value, %	12.6	T 0316
	Los Angeles abrasion, %	29.8	T 0317
	Water absorption, %	0.32	T 0304
Fine aggregate	Specific gravity	2.758	T 0328
	Sand equivalent, %	70.2	T 0334
Mineral powder	Specific gravity	2.655	T 0352

**Table 3:** Physical properties of SBS-modified asphalt.

Test Items		Test Results	Test Method [21]
Density, 25 °C, g/cm^3^		1.029	T 0603
Penetration, 25 °C, 0.1 mm		44.7	T 0604
Softening point, °C		71.8	T 0606
Ductility, 5 °C, cm		36.7	T 0605
Viscosity, 135 °C, Pa·s		2.13	T 0625
RTFOT	Mass change, %	0.53	T 0604
	Penetration ratio, 25 °C, %	81.4	T 0606
	Ductility, 5 °C, cm	27.5	T 0605

**Table 4:** Basic performance of rejuvenator.

Test Items	Test Results	Test Method [21]
Viscosity, 60 °C, mm^2^/s	108	T 0619
Flash point, °C	231	T 0633
Saturates content, %	26.2	T 0618
Aromatic content, %	30.3	T 0618
Mass change before and after thin film oven test, %	−2.6	T 0609

**Table 5:** Physical properties of two composite-modified asphalts.

Test Items	SBS-HVA	SBS-CRM	Test Method [21]
Density, 25 °C, g/cm^3^	1.047	1.048	T 0603
Penetration, 25 °C, 0.1 mm	42.5	46.7	T 0604
Softening point, °C	86.4	82.8	T 0606
Ductility, 5 °C, cm	25.3	18.4	T 0605
Viscosity, 135 °C, Pa s	5.45	20.5	T 0625

**Table 6:** Marshall index test results of different types of asphalt mixtures.

Mixture Types	Test Items	Asphalt Content, %		
		3.8	4.3	4.8
SBS-V	Specific gravity	2.389	2.413	2.430
	Air voids, %	6.42	4.61	2.91
	VMA, %	13.60	13.20	13.04
	VFA, %	52.77	65.06	77.70
	Marshal stability, kN	13.01	12.87	12.36
	Flow value, mm	2.08	2.07	2.13
SBS-R	Specific gravity	2.379	2.410	2.433
	Air voids, %	6.07	4.13	2.47
	VMA, %	13.97	13.30	12.92
	VFA, %	56.55	68.99	80.92
	Marshal stability, kN	13.64	14.45	14.34
	Flow value, mm	2.07	2.11	2.13
SBS-HVA-R	Specific gravity	2.369	2.396	2.417
	Air voids, %	6.65	4.87	3.31
	VMA, %	14.32	13.80	13.48
	VFA, %	53.59	64.85	75.55
	Marshal stability, kN	19.85	19.24	20.83
	Flow value, mm	2.18	2.80	2.99
SBS-CRM-R	Specific gravity	2.331	2.368	2.410
	Air voids, %	7.54	5.97	3.59
	VMA, %	15.71	14.80	13.74
	VFA, %	52.02	59.66	73.85
	Marshal stability, kN	17.69	19.42	20.93
	Flow value, mm	2.60	2.46	2.23

**Table 7:** The growth rate of water absorption under different scouring cycles compared to the 0 cycles’ number.

Mixture Types	Growth Rate, %			
	1000	3000	5000	7000
SBS-V	434.7	646.9	706.1	861.2
SBS-R	304.1	485.1	552.7	594.6
SBS-CRM-R	353.0	566.7	568.2	656.1
SBS-HVA-R	251.5	521.2	556.1	656.1

**Table 8:** The growth rate of air voids under different scouring cycles compared to the 0 cycles number.

Mixture Types	Growth Rate, %			
	1000	3000	5000	7000
SBS-V	7.4	13.3	20.5	20.7
SBS-R	9.7	34.4	34.6	36.9
SBS-CRM-R	7.1	15.4	15.6	31.5
SBS-HVA-R	9.2	21.9	29.5	33.1

**Table 9:** The decline rate of splitting tensile strength under different scouring cycles compared to the 0 cycles number.

Mixture Types	Decline Rate, %			
	1000	3000	5000	7000
SBS-V	4.9	9.8	11.9	22.1
SBS-R	12.4	21.6	28.8	33.3
SBS-CRM-R	19.1	27.0	32.4	37.2
SBS-HVA-R	2.6	4.8	6.8	12.3

**Table 10:** Test results of low-temperature crack resistance performance indicators for different types of asphalt mixtures.

Mixture Types	Peak Load, kN	Fracture Energy, J/m^2^	Fracture Toughness, MPa/m
SBS-V	8.89	794.2	1.360
SBS-R	7.87	642.8	1.202
SBS-CRM-R	8.12	735.4	1.241
SBS-HVA-R	8.35	715.3	1.226

## Data Availability

The testing and analysis data used to support the findings of this study are included within the article.
